# Adult education and entrepreneurship: getting young adults involved

**DOI:** 10.1186/s13731-023-00277-0

**Published:** 2023-03-09

**Authors:** Isaac Kofi Biney

**Affiliations:** University of Ghana, Legon, Accra, Ghana

**Keywords:** Entrepreneurship, Adult education, Human capital theory, Growth and enterprising mindsets

## Abstract

This paper reflects on adult education and the fostering of an entrepreneurial mindset. It solicits roles adult education, especially the non-formal education (NFE), could play in fostering entrepreneurial mindset of young adults. It examines short-to-medium, and long-term plans of young adults in nurturing growth and enterprising mindsets through involvement in NFE endeavours. It probes into entrepreneurial opportunities and challenges in the communities that young adults could recognise and create enterprises for themselves. This is a narrative case study which purposively selected, as the unit of analysis, an adult learner who was operating a micro-enterprise and pursuing a degree programme at Accra Learning Centre. An in-depth telephone interview was conducted to garner stories and experiences young adult have had innovating with an entrepreneurial mindset. Thematic, analytical, narrative and interpretivist approaches were adapted in presenting the results. The participant had a good experience in his start up, he learned lessons, and worked hard to grow his micro-enterprises. The savings culture he built allowed him to cope with difficulties posed by Covid-19 pandemic to his micro-enterprises. Governments and stakeholders in entrepreneurship should via policy, advisory and financial support motivate young adults to invest in entrepreneurship and create sustainable jobs for themselves.

## Introduction

Globally, attention is being placed on entrepreneurship amongst young adults because they appear especially vulnerable to unemployment (Baah-Boateng, 2018). Projections for Africa indicate that the proportion of young adults will grow by 2035 (Dadzie et al., 2020), and this presents both opportunities and challenges. In Ghana, for instance, the youth make up 36 percent of the population and 56 percent of them live in the urban areas (Dadzie et al., 2020). Similarly, Takyi-Appiah (2022) opines that Ghana is a young country with the vast majority of the population under 36 years old and needs to find a way to inspire and drive young adults so that they can harness the massive potential that Ghana holds and boost development. Meanwhile, entrepreneurship is a major engine for economic growth and job creation (Acs, 2006; Nafukho & Muyia, 2010; Wong et al., 2005). Entrepreneurship learning is the basis for creative and innovative ideas to succeed in the twenty-first century (Lindner, 2020). Entrepreneurship empowers young adults to create sustainable ventures and combat high unemployment, especially among university graduates in African universities (Biney, 2018a, 2019, 2021; Nafukho et al., 2011). The youth represents a large population of potential entrepreneurs (Biney, 2018a; Gruidl & Markley, 2015); and one way of integrating more young people into the labour market is to increase youth entrepreneurship (Biney, 2018a; Green, 2013). Thus, enterprise education can enhance student employability to be opportunity-focused, self-aware, and attuned to the business environment (QAA, 2012). Many young adults see entrepreneurship as the ideal way to create their own job security and career success (Scarborough, 2012); yet the scientific study of youth entrepreneurship is in its infancy (Geldhof et al., 2014).

Hence, fostering *growth* and an *entrepreneurial* mindset has the potential of making young adults become self-employed. Over the years, entrepreneurship has been linked to a myriad of the most pressing and compelling topics and themes of its day (Audretsch & Moog, 2020); ranging from job creation (Birch, 1981; Storey, 1984), to innovation (Lerner, 2012; Lindner, 2020), pathway to economic growth and productivity (Acs, 2006; Clifton, 2011; Foster et al., 2008), social goals (Saebi et al., 2019), and the inclusion of socially and economically excluded people and communities (Hughes et al., 2012; Lyons, 2015). More so, the noteworthy contribution of entrepreneurial activities to economies (Keilbach & Sanders, 2008, cited in Daim et al., 2016) in terms of growth, innovation, job creation, and poverty reduction (Lunati et al., 2010, cited in Daim et al., 2016) makes entrepreneurship a popular research topic. However, going through the literature, it was clear that very little has been written on adult education and entrepreneurship. Meanwhile, humanistic adult education philosophy tends to support entrepreneurialism (Zinn, 1999). Thus, people learn to innovate to create ventures and add values to themselves. The aim of this paper is to fill the gap that adult education can be linked to entrepreneurship since both fields involve learning.

The NFE is perceived as any organised, systematic educational activity, provided within the classroom or outside of the framework of the formal system, whatever its purposes, target groups and providers (Peace Corps, 2004; Singh, 2010). To Peace Corps (2004), NFE means adult education; and includes short entrepreneurial training programmes, workshops, seminars, symposia and management boot-camps. The skills acquired are for immediate application in day-to-day life, not as with most formal education to prepare for some later purpose (Rogers, 1993). Yet, this narrative paper on adult education and entrepreneurship used an adult learner pursuing a degree programme by distance education (DE) mode. It argued that when an entrepreneurial mindset is fostered in young adults even as they learn formally in the schools, colleges and universities, they would also learn non-formally and informally in the world of work to become innovative in their entrepreneurial drive. After all, an entrepreneurship movement has advanced into higher education (Wright et al., 2022).

Again, the short-to-medium, and long-term plans young adult learners put in place while fostering an entrepreneurial mindset could serve as pathways to becoming entrepreneurial in thinking and action. According Rasmussen (2014), and Debyser (2013), the European Union (EU) has always indicated that adult education has a strong link to work and the labour market; adding that the *“New Skills for New Jobs initiative”* adopted by the EU is designed to improve employment by supporting *entrepreneurship* and *self-employment* (p. 29). The United Nations, Educational, Scientific and Cultural Organisation (UNESCO, 2015), and Knowles et al., (2020) see adult education as a multidisciplinary process oriented to favour lifelong education for all, and efficient learning throughout life. Adult education seeks to inform learners about how to be *subjects* in their lives, and how to make decisions that strengthen their opportunities to be active and improve society (Freire, 1973). It aims at providing knowledge that improves professional qualifications and to achieve civic, social, moral and cultural attitudes and skills for performing responsibilities and for progress in all spheres of life (UNESCO, 2015). Thus, adult education is working *with* adults to *promote* learning *for* adulthood. The purpose of adult education is to prepare individuals to be responsible citizens socially, economically, and politically (Oduro-Mensah, 2009). Adult education improves access through more flexible pathways to learning for low skilled and vulnerable workers (Rasmussen, 2014), and it is virtually impossible to talk about adult education and not refer to the world of work (Biney, 2022; English & Mayo, 2012). This presupposes that when young adults invest their time engaging in NFE by way of attending workshops, seminars, and symposia on entrepreneurship, they can put into practice skills, knowledge, attitudes, and values of entrepreneurship to create jobs and become innovative in their communities.

According to Ofori-Atta (2021), about 16 million people joined the workforce yearly across Africa, with 83% of them unable to find jobs, and as a result of which the unemployment rate kept increasing every year. For instance, Ghana’s unemployment rate has hit 13.4% in 2021, up from 5.3% in 2010 (Daily Graphic, 2022). In Ghana, today, even the formally educated young adults have difficulties securing jobs because of paucity of formal job opportunities. The state enterprises built in the 1960s and 70 s have collapsed due to poor management and maintenance culture. The new initiatives by the Government of Ghana (GoG), including *‘one district one factory’*, *‘planting for food and jobs’, ‘planting for export and rural development’, ‘rearing for food and jobs’* are yet to make an impact in developing job opportunities for young adults. More so, the GoG industrialisation drive to support existing industries and the private sector players to create new industries is not moving fast enough to create more job opportunities for the inceasing number of young adults. Many young adults, especially those living in the mining communities, have resorted to illegal mining activities popularly known as ‘galamsey,’ meaning, ‘gathering of mineral resources, including gold for sale.’ As Martinez-Martinez et al. (2022) observe, in a world in which the environment is more deteriorated, it is important to be aware of the advance in environmental knowledge to take care of it and eliminate environmental degradation. In the Ghanaian situation, however, environmental degradations have created huge challenges, including the destruction of water bodies and forest vegetation cover, and the wider environment. Coupled with these difficulties is the fact that, many more young adults are also participating in the distance education (DE) mode of learning (Biney, 2021). When such young adults are introduced into entrepreneurship and supported to practice it, they would reduce the increase in the number of unemployed university graduates in Africa. The Accra Learning Centre of University of Ghana has over 11,000 adult learners on its DE programme, and there are other dual-mode universities, and private universities that turn out graduates every year to the labour market.

The questions are: How many of these educated young adults would like to follow a career as an entrepreneur? If many are not interested, what could have been the reasons for that? Is it the case that young adult learners cannot take an informed risk? If yes, what can facilitators do to foster entrepreneurship in such students to become entrepreneurial in their intentions and actions? These questions have to be answered if developing countries are to realise development in terms of job creation for young adults. Lackeus (2015) asserts that the *how* to make students more entrepreneurial is probably the most difficult question, and this is what this study probes into. Harkema and Popescu (2015) aver that the number of students that decide to follow a career as entrepreneur is low in the Netherlands compared to the United States of America, another advanced country. They explained that, it could partly due to national cultural aspects such as a risk avoiding attitude and uncertainty avoidance which does not accept failure easily. A study by Kauffman Foundations shows that the level of entrepreneurial activity is higher among people age 55 to 64 than that among people aged 20–34 (Scarborough, 2012). On the contrary, entrepreneurial potential among young adults seems higher than older adults in Ghana (Biney, 2021, 2022). Lyons (2015) adds that he has long been an advocate of entrepreneurship as a mindset, process, skill set, and tool kit that can help us solve economic, environmental and social challenges that communities face. However, it can be argued that continuous learning and adaptation largely aid us to meet the needs and opportunities that dynamically emerge from daily situations (Bolisani & Bratianu, 2017; Cegarra-Navarro et al., 2010). Yet, many researchers claim that the only way to make people entrepreneurial is by applying a *learning-by-doing,* thus, apprenticeship approach (Biney, 2021; Lackeus, 2015), but the question of doing *what* needs to be answered. However, “learning-to-create value” has been grounded in *entrepreneurship* (Lackeus, 2015); and entrepreneurial opportunities exist in the communities which young adults can tap into and make themselves useful. This study explored the importance of adult education and entrepreneurship by examining *how* young adults could be supported to get involved in adult education programmes as a precursor to impacting upon entrepreneurship. In so doing, the adventurous young adults can create job opportunities for themselves and assist many young adults to become employed, stay in their communities and make those communities vibrant and sustainable. This study purposively explores adult education and the entrepreneurial drive amongst young adults, and is guided by seven research questions presented below.

## Research questions

1. When and how did you start fostering entrepreneurial ideas and mindset?
2. How has your startup fared in the past two years?
3. Where do you intend to take your micro-enterprise to in five years’ time?
4. Between five to ten years’ time where do you think your enterprise would be?
5. Is participation in NFE programmes part of your plans of growing your enterprise?
6. Which of the NFE ideas have you been utilising to grow your enterprise?
7. Are there challenges facing your enterprise, and how have you been coping?

Based on my interest in youth entrepreneurship and observations made, for example, watching, listening and readings from dailies, television, radio and Government of Ghana’s website, Springer, Routledge (Taylor & Francis), ERIC and Sage journals on the state of youth unemployment, I became informed about research questions to pose to the participant. The findings from such a study could help to fill research gaps of making today’s young adults entrepreneurial to create ventures and become self-employed. Merriam et al. (2019) argues that research questions emerge from the researcher’s interests and observations made on a novel or puzzling phenomenon under-study in a particular situation.

Similarly, I reflected upon the potential of adult education and entrepreneurship to young adult learners to foster entrepreneurial skills and mindsets and become self-employed. Hence, motivating young adults to learn and acquire enterprising skills and mindsets through education, training, and mentorship is crucial today due to mass-extensive unemployment problem confronting young adults globally, including Ghana. I argue that timing and appropriate entrepreneurial training programmes young adults avail themselves of, and benefit from, put them in a better stead to foster enterprising mindsets, take informed risks to innovate, and create sustainable ventures for themselves. As a budding entrepreneur, one must make participation in short training programmes aid him or her in evaluating the success or otherwise of venture created. Such evaluation exercises could help budding entrepreneurs to undertake growth projections of their enterprises in the short, medium and long-term basis.

Even as there are training programmes being run for small-business operators, prospective venture operators must always participate in training programmes that add value to and empower them to manage their enterprises on sustainable and profitable lines. Thus, when there is a pandemic such as Covid-19, such small-business operators could still operate and manage their enterprises on profitable and sustainable lines.

## Theoretical framework

This study is framed by adult learning and human capital development promoted by United Nations Educational, Scientific, and Cultural Organisations (UNESCO, 2010), Knowles et al. (2020), Singh (1999); Unger et al. (2011), English and Mayo (2012), and Hong and Crowther (2016). It also connects with Carayannis and Campbell’s (2010) works on quadruple helix and quintuple innovation helix concepts; and is an interdisciplinary and trans-disciplinary study. The researcher attempts to relate adult education to entrepreneurship in building appropriate human capital in fostering entrepreneurship. The point is that when young adults learn to develop an entrepreneurial eye to recognise unmet needs and opportunities in their communities, they can become empowered to innovate and exploit those opportunities in their communities. In so doing, they add value to the start-ups created. As many young adults are engaged productively in ventures, they would not have the luxury of time to indiscriminately degrade the environment by engaging in ‘galamsey’ activities, with negative ramifications. Thus, the knowledge young adults might have acquired would stimulate them sufficiently to protect the environment to engender sustainable development. After all, the quadruple helix blends in the perspective of a media-based and culture-based public, and the quintuple helix frames knowledge and innovation in the context of the environment (natural environments) (Carayannis & Campbell, 2009). Indeed, the Quintuple Helix thus offers an analytical frame where knowledge and innovation, are being connected with the environment (Carayannis & Campbell, 2010).

To UNESCO (2010, 2022), English and Mayo (2012), the community-based political and cultural traditions of adult education promoted by Freire and Nyerere in the 1960s were challenged by the introduction of adult education policies as a means for *economic development* framed within the notions of human capital. UNESCO (2022) argues that there is the need for young adults to foster lifelong learning mindseta, because there is a growing demand for advanced skills, creativity and adaptability in the workplace all due to the market transformation. These skill sets include entrepreneurship.

Similarly, Hong and Crowther (2016) discussed Scottish experiences on learning and entrepreneurship perspective policy contexts in community education that focused on *entrepreneurship and employability*. Singh (1999) was of the view that educating adults meant contributing to their self-reliance and personal economy to exercise their basic rights, and to increased productivity and labour efficiency. Adult education, to the practitioners, is a “human resource and productive investment” and must be utilised as such (Singh, 1999, p. 3). Therefore, investment in adults’ acquisition of an entrepreneurial mindset is critical as Sernau (2009) asserts that the United States (U.S.) education system with its more flexible structure and emphasis on choice and creativity has promoted a culture of innovation and entrepreneurship. It is not surprising that the major goals of adult education include “preventing and combating unemployment, promoting a smooth transition from school to work, social inclusion and social cohesion” (Singh, 1999, p. 3).

The term human capital first coined by Adam Smith in 1776 was promoted by Theodor Schulz and Gary Becker, stating that “investment in education and skill formation is as significant for economic growth as investment in machines and equipment” (UNESCO, 2010, p. 22). Unger et al., (2011, cited in Ensign & Farlow, 2016) used the human capital approach to discuss habitual entrepreneur (experienced entrepreneur), to examine the correlation between task relatedness (vis-à-vis the accumulation of entrepreneurial experience, knowledge, and skills), and entrepreneurial outcomes. English and Mayo (2012) assert that human capital theory is sociologically considered as an offshoot of structural functionalism based on the overstated consideration that the more one invests in one’s education the greater the economic return. Not surprising, Nguyen et al. (2021) argue that human capital has a moderating effect in reducing the negative impacts of weak institutions.

Ensign and Farlow (2016) theorising on habitual entrepreneurs in their paper *‘serial entrepreneurs in the Waterloo ecosystem’*, discussed an entrepreneurial ecosystem as a holistic view which seeks to understand the institutions and structures (formal or informal), that help to develop and maintain entrepreneurial human capital (Baron & Markman 2005, Davidson & Honig 2003, Lengyel et al. 2015; Mason & Brown, 2014; Napier & Hanson, 2011; Westlund & Bolton, 2003, cited in Ensign & Farlow, 2016). To them, the ecosystem helps entrepreneurs build social capital because of the abundance of individuals involved in entrepreneurship. In effect, the entrepreneurs have the opportunities for networking and the exchange of ideas within the ecosystem. For adult education and entrepreneurship, both fields involve learning; hence, young adults learn formally in the Universities and informally and non-formally from experienced entrepreneurs, media outlets, and apprenticeship and mentorship roles they go through to acquire hands-on experiences in entrepreneurship. It is further argued that highly educated people can do more and do it better, and contribute more to national development (Sernau, 2009); yet, investment in education alone cannot guarantee increased productivity at workplace, unless other variables come into play.

Supporting this observation, Naude (2013) indicates that improving the quality of entrepreneurial ability does not imply only improving the *skills* and *education* of entrepreneurs, their ‘human capital’, but focusing on the *innovative* abilities of entrepreneurs. That is to say, for education to become a true investment in human capital that will offer a return in productivity, it must be connected to a viable means of application. Thus, fostering entrepreneurial mindsets amongst young adults is crucial considering the massive levels of unemployment confronting them; hence, effort has to be made to connect education with opportunities in our communities for productive application. This study is important, because adult education is being matched to address the critical issue of unemployment in developing countries, including Ghana.

The more a country invests in education without commensurate investment in complementary economic structures, the more likely it is to have a surplus of educated and skilled persons’ who are unemployed. When such a situation arises, according to Agyeman (1993), school leavers are compelled to reduce their expectations about the kind of work they would get and to come several notches down their scale of preferences for employment, adding that such crises between education and the economy were not limited to countries in Africa alone, but India and Pakistan had similar experiences, where university graduates have to take jobs as bus drivers and ticket sellers. Today, this phenomenon appears to be the case of young adults in Ghana, making their situation a bit precarious; hence, fostering an entrepreneurial mindset in young adults seems the best way forward. After all, Sen (1999) brought a new twist to the discussion of the theory of human capital by focusing on the *capability approach* to development. Sen (1999) states that “enhancement of human freedom is both the main and the means of development; thus, people have to be actively involved in *shaping* their own destinies, rather than the passive recipients of development programme” (cited in UNESCO, 2010, p. 23). Similarly, Dweck (2008, 2016) opines that people have more capacity for lifelong learning than they ever thought, adding that experience, training, and personal effort take them the rest of the way. Hence, encouraging entrepreneurship within our communities as observed by Gruidl and Markley (2015) [through adult education], is one of the most effective development strategies in the developing countries. Even community developers, have largely come to appreciate that entrepreneurship is crucial to the vitality of the rural communities (Gruidl & Markley, 2015). Therefore, the culture of entrepreneurship is to be created in young adults in the developing countries to *innovate* and create ventures with value-additions, and make their communities more thriving and sustainable.

## Literature review

This paper reviews literature in related areas including adult education for entrepreneurship; growth and enterprising mindsets of young adults; and entrepreneurial opportunities and challenges in the communities.

### Adult education for entrepreneurship

In many countries, adult education has been utilised to bring in equitable distribution of the opportunities of society (Singh, 2010). The NFE that many practitioners assert is adult education, is any organised, systematic educational activity carried within the classroom (Singh, 2010); or outside the formal education system to provide selected types of learning to adults and children (UNESCO, 2010). In the twenty-first century all individuals need to develop the capacity to be creative and innovative at work and in their communities, because the world of work is undergoing major transformations caused by technological change, shifts in the global economy, new business models, and demographics (Lindner, 2020). Not surprisingly organisations like UNESCO, the European Commission, the OECD (2018), and the ILO (2019) recognise the importance of enabling entrepreneurship as a way to drive growth. I argue that this perceived growth in entrepreneurship is to build the capacity of young adults to become self-employed. After all, young adults are often the first casualty when redundancy is declared at workplace (Baah-Boateng, 2018). However, this step can be realised when education and training of young adults is placed high in Ghana’s developmental drive agenda. Another observation is that sustainable enterprises are a priority for the whole education system (Lindner, 2020); including adult education.

Entrepreneurial training (King, 2017), organisation of workshops, seminars, and symposia on small businesses in the communities (Biney, 2017, 2018a, 2018b, 2019, 2021), business training and start-ups (Nafukho et al., 2005; Peace Corps, 2004), management training and enhancement of confidence (Jonsdottir, 2006), and farmer training programmes and adult literacy programmes and occupational skill training (UNESCO, 2010) to young adults constitute some examples of NFE. Youth clubs with educational purposes, and community programmes of instruction in health, nutrition, family planning, cooperatives and [entrepreneurship] are also NFE programmes (UNESCO, 2010).

Therefore, the NFE is a deliberate process of communicating ideas and developing skills in adults to participate more intelligently in economic and civic programmes to achieve other personal and social goals (Case & Niehoff, 1976). Unsurprisingly, Nafukho and Muyia (2010) argue that students who have taken an entrepreneurship course have learned to be creative and innovative, and should seek to be employment creators and not job seekers. However, such trained students can only be successful in their entrepreneurial drive when they continue to participate and get involved in short training programmes such as entrepreneurship workshops, seminars, and management boot-camps to build on their already acquired knowledge, skills, values and understanding. Such training programmes are NFE, and are practically oriented. The knowledge and skills acquired in the training programmes are immediately applied to solve problems. This supports Singh’s (2010) assertion that NFE focuses on the learner’s needs, and uses the learner as a resource, and stresses on relevant activities and practical outcomes. In these difficult times of the global COVID-19 pandemic, the issue of unemployment has become topical. The pandemic has created disruptions in organisations, creating massive unemployment, particularly amongst young adults.

This unemployment problem is severe amongst young adults in the developing countries, including Ghana. In Ghana, young adults between 15 and 35 make up about a third (36 percent) of Ghana’s population are largely unemployed (Dadzie et al., 2020). Hence, encouraging young adults into adult education by the NFE process and to invest their funds and become involved in entrepreneurship is one good step of becoming self-employed to create job opportunities for many others in our communities.

### Growth and enterprising mindsets of young adults

These two concepts in entrepreneurship, thus, *growth* and *enterprising* mindsets, should be watchwords for budding entrepreneurs, and young adults must be conversant with them. Carol Dweck, a developmental psychologist and professor at Stanford University, coined the two terms, growth and fixed mindsets. She stated that mindset in general refers to implicit theories about the malleability of human characteristics (Dweck, 2008, 2016). Thus, our personal characteristics can be changed; and this demands that we continue to learn. In many instances, entrepreneurial characteristics can be learned; however, some think that personal characteristic are fixed and cannot be changed (Knowles, 2003). Indeed, people with a growth mindset have certain characteristics that with *training* and *practice* we can improve and change (Zappe, 2019). On the contrary, if we have a fixed mindset characteristic, we believe that training, practice, or experience will have no effect on those characteristics. Since approaches to fostering, creating and managing enterprises are not static but dynamic, learning about changes taking place in entrepreneurial activity is important. This provides ample credence that engaging in adult education in the form of participating in short seminars, workshops, and management boot-camps are critical in charting a path to becoming an entrepreneur in today’s digital global economy. Learning and technology are of essence in this fast-paced business environment.

Creativity in thinking that results in new ways of solving problems constitutes a critical part of fostering an enterprising mindset (Cordeiro, 2007). In fact, utilising both left and right halves of the brain, and also thinking in convergent and divergent ways are critical in developing oneself as an entrepreneur. No wonder, Jones (2015) argues that people with an enterprising mindset have a high need for self-fulfillment, a passion to create something so that others benefit, the desire to make their ideas become reality even when things get in the way, and are self-motivated. Kourdi (2015) describes it as pragmatism in thinking; however, Murphy (2010) avers that not everyone with an enterprising mindset wants to become his/her own boss. This observation is supported by Talmage and Gassert’s (2020) assertion that entrepreneurship must challenge students to better identify the social impacts of enterprises and innovations in addition to economic impacts. Zappe (2019) asserted that the entrepreneurial mindset is a set of attitudes, skills, and behaviours that help students to succeed academically, personally, and professionally; and includes initiative and self-direction, empathy, risk-taking, flexibility and adaptability, creativity and innovation, critical thinking and problem solving.

### Entrepreneurial opportunities and challenges in the communities

Places where people live have profound effects on their economic and social outcomes (Brown & Baker, 2019); and entrepreneur development is a place-based activity (Markley et al., 2015). Indeed, where community exists it confers upon its members’ identity a sense of belonging, and a measure of security. Thus, entrepreneurship can help confer identity, belonging and security not only on those who elect to start and grow enterprises, but also on those who join them in their effort, and on the wider environment in which they operate (Gardner, cited in Smilor, 1997). There are micro entrepreneurial opportunities for young adults in the communities, such as batik tie and dye, fruit juice production, uber transport business, oil palm processing, soap making, pomade and powder making, cream production, bakery and cassava processing among others. However, it takes people with entrepreneurial eye to identify an unmet need in a community. Many of such entrepreneurial ventures often start as meso, micro, small, medium, and then large ventures.

Frank and Smith (1999) observed that community members in a small community in Canada were disturbed by the fact that many of their young people were going to larger conurbations to find work, and as a result of this concern, and after much work, some business people sponsored a small local sawmill. This mill was a success, and other business opportunities were considered to help create additional jobs in the community. The point is that when entrepreneurs see one venture succeed, it is easier for them to plan others. This demonstrates that the success of cultivating growth and enterprising mindsets of young adults in the communities depends, to a large extent, on the degree of active and willing involvement of young adults to nurture the basics of entrepreneurship. Doing so means that young adults develop an entrepreneurial eye to recognise needs in their communities and exploit them through sustainable ventures. After all, the pivot of life of every man or woman is a job, and if young adults are to have any degree of social security, they must develop a self-reliant spirit to create start-ups in their communities. Similarly, taking informed risk, innovating, planning and managing resources, researching the market, and developing passion to make a difference in other peoples’ lives by participating in seminars and workshops to build capacity is critical in becoming entrepreneurial in thinking and action.

However, there are challenges start-ups in every economy face as budding entrepreneurs attempt to drive their entrepreneurial ideas. Cordeiro (2007) asserts that more than 70% of newly established small enterprises tend to collapse in their first year. Legodi and Kanjere (2015) identified lack of skills as a challenge facing a majority of enterprise promoters. To succeed as an entrepreneur requires *continuing education* and *training* which many young entrepreneurs do not involve themselves in (Biney, 2019, 2021). Many youth-led enterprises seem to lack financial literacy and cannot take informed risks to grow their start-ups (Biney, 2018a, 2019, 2021). Inadequate access to finance; lack of financial information and business support services from banks (Amadasun & Mutezo, 2022); lack of training and lack of managerial talent are challenges facing entrepreneurs (Abor & Quartey, 2010; Hameed & Irfan, 2019). Insufficient commitment to drive start-ups successfully has been identified as another reason of individuals to fail in entrepreneurial abilities (Schermerhorn, 2005). Lack of communal realisation about new entrepreneurs and companies (Hameed & Irhan, 2019); and fear of failure (Tan & Ng, 2006) are further challenges facing entrepreneurs.

## Methods

This section provides information on the methods employed to conduct the study. It begins with the participant information, and followed by research design, sample, and sampling strategies to recruit the participant. Data collection techniques, and finally, data analysis follow in that order.

### Participant information

A first year (Level 100) adult learner pursuing a degree programme by distance education (DE) mode at Accra Learning Centre (ALC), and operating a printing shop as a micro-enterprise served as the unit of analysis of the study.

### Research design

The study adopted a narrative case study design and was conducted at ALC. The narrative design the researcher adapted locates the study in the qualitative research paradigm. Philosophically, this narrative case study is a form of narrative inquiry based upon the constructionist view of social reality, ideas and practices. The researcher holds the view that human social life is based less on objective, hard factual reality than on ideas, beliefs, and perceptions that people hold about reality. Thus, stories of lived experiences were co-constructed and negotiated between the researcher and participant as a means of capturing complex, multi-layered and nuanced understandings (Etherington, 2013). In this narrative case study, then, it is about *how* the participant fostered and applied an entrepreneurial mindset. The study, therefore, used thematic, narrative interpretivist approaches of the participant’s experiences and stories garnered from learning in higher education institutions (HEIs). In addition, participation in NFE programmes including workshops, seminars, symposia and management boot-camps on entrepreneurship in which participant engaged in were also solicited. Some interpretivist approaches were adapted, because the study sought to provide thick, rich descriptive data (Creswell & Poth, 2018).

### Study sample and sampling techniques

The researcher adopted a purposive sampling procedure, using the snow-ball approach to select the participant for the study. Through the ALC DE Student Representative Council (SRC) president, the researcher located a student possessing the characteristics required for the study. The researcher intentions was to capture the detailed inner world of the participant experiences in pursuing a higher education programme and operating a printing shop, and learning about the enterprise through NFE programmes. As a qualitative researcher, and conducting a narrative case study research type, I knew the kind of information I was looking for, and found the participant most appropriate for the study. However, this narrative study is guided by the theory of *human capital* lens, and undergirded by the seven research questions stated above.

### Data collection

Narrative studies focus specifically on *stories* told by individuals; and for narrative inquirers, experience is the stories that people live and tell over time in different places, and in diverse and unfolding relationships (Clandinin et al., 2019). Indeed, Clandinin et al. (2019) avers that narrative explored through interpretive research allows access to the respondent reality via the socially constructed stories. This makes narrative study both a *method* and *the phenomenon.* As a method, it begins with the experiences as expressed in lived and told stories of individuals, and this study is a narrative case study, which uses *narrative* as a method, phenomenon, and analysis of the results. Detailed stories and experiences of the participant on how he nurtured entrepreneurial mindset, the NFE programme participated in, online exploration undertaken in managing printing shop, progress made in entrepreneurial drive, challenges encountered and coping strategies adopted were presented and interpreted; bearing in mind that stories are *reconstructions* of the participant experiences.

Narrative inquiry is used to study *educational experiences* since it is argued that humans are storytelling organisms who lead storied lives (Savin-Baden & Niekerk, 2007), and as adult educator with a research interest in entrepreneurship, I often facilitate learning using stories, hence this approach appears convenient for the study. The participant was assured of anonymity, and his consent was sought before undertaking the in-depth telephone interview. The researcher adapted Hollway and Jefferson’s (2000) four principles which facilitate the production of the interviewee’s meaning; and ensured that open-ended questions were mainly posed. The seven questions sought to elicit experiences and stories from the participant. Since *why* questions sought to encourage intellectualization, and can be threatening, I avoided them. Probing questions, using participant ordering and phrasing were posed to the participant.

### Data analysis

Since procedures of qualitative narrative inquiry consist of focusing on studying *one* or *two* individuals, gathering data through the collection of their stories, reporting individual experiences, and chronologically ordering the meaning of those experiences (Creswell & Poth, 2018); made the researcher adapt to the best practices in analysing rich and relevant experiences or data garnered for this study. As a qualitative researcher, I kept field notes of participant experiences to aid the analysis of results. I listened effectively to the participants’ stories, and acknowledged mutual construction of the narratives between the researcher and the participant. I also ensured that the stories created by the participant were recreated, and negotiated. Thus, both the participant and researcher were co-creators and interpreters of the narratives to make the experiences of the participant meaningful. In analysing the stories and experiences shared by the participant, I first examined the epiphanies and metaphors inherent in the stories and experiences to extend and illuminate the meanings constructed. This approach aided the researcher to secure meaningful stories and experiences from the narratives. The results were presented in the first person, and since the stories collected were many, I summarised them to make them more meaningful. This approach adopted helped me to interpret the data by focusing on the participant context.

Since the paper was a narrative case study type, I took a cue from qualitative researchers such as Merriam et al. (2019), and Creswell and Poth (2018) in analysing qualitative data gathered. First, I read thoroughly the raw data to identify initial themes that emerged. Second, I built a thematic framework made up of themes and sub-themes after identifying general patterns at the first stage. At the next stage, the themes that were identified were indexed by assigning the same numbers to themes that had similar interpretations that allowed for proper categorisation of thematic charts to synthesise the data. The researcher developed the data into broader categories and themes to form a fuller picture of participant experiences. Interpretations and meanings were given to the data gathered. The use of *‘stories’* has been employed in this study, and this step is followed by an interpretivist analysis of the themes where elements were properly refined by inspecting each column of the thematic chart across all cases to identify the content and dimensions of each case. This ensured a better refinement of the various categories that were identified. The next stage searched for patterns and links between sets of phenomena and between the participants’ stories and experiences narrated. This stage involved associative analysis. The final stage involved a discussion of the findings of the study in the context of existing literature. In-depth scrutiny of the data was undertaken by the researcher to arrive at the final themes and sub-themes. Thematic analysis was performed on the data, and the results of the study are presented next.

## Results

This section is divided into seven sections based on themes derived from the narrative data garnered. The results are presented in narrative style of *stories*, *experiences,* and *meanings* negotiated between the researcher and the participant. The participant was a 26 year old first year (Level 100) male student pursuing Bachelor of Arts degree programme, reading Human Resource Management, Psychology and Information Studies. The participant operates a printing shop in the form of micro-enterprise to cushion him to pursue degree programme by DE mode at Accra Learning Centre. The results of the study were presented under the following themes: (1) entrepreneurial ideas and mindset, (2) progress in micro-enterprise in the short-term, (3) prospects of micro-enterprise in the medium term, (4) state of micro-enterprise in the long-term, (5) participation in NFE and informal programmes, (6) preferred NFE/informal programmes to grow micro-enterprise, and (7) challenges and coping strategies.

### Entrepreneurial ideas and mindset

On how and when the participant started fostering entrepreneurial ideas and mindset, he chronicled events leading to nurturing entrepreneurial mindset, and presented it thus:

I completed Senior High School (SHS) in 2014, but due to financial challenges, I could not further my education immediately to tertiary education level. One unfortunate difficulty I faced in September 2009 was that I lost my mother, and that was a contributory factor which delayed my tertiary education because, financial-wise, my dad was insolvent and could not support my education. My father had five wives with eleven children. The little money he made is dissipated to the five wives and the eleven children, making it difficult to fund my education at the tertiary level. You know, Mr. Interviewer, I’m a twin brother, and many of my siblings truncated their education very early due to this challenge. I’m even lucky to have come this far, because there was no hope for my tertiary education. Thank God, following a friend’s intervention in 2016, I came up with an idea of a start-up. This is how and when I started fostering entrepreneurial ideas, and entered into printing shop business. One day, I went to a friend to accompany him to do a photo-copy of a manuscript. Over there, I saw people coming in and out of the printing shop to do photo-copy, bind and laminate learning materials. The business environment was inviting, and three workers were engaged doing different types of work. The first was into photo-copying, the second into binding, and the third into laminating of documents. The manager of the printing press was engaged in computing and design works. He was designing notices, obituaries, wedding brochures, flyers among many learning materials. I interviewed the manager for insight into the business, including the cost of the machines used, and the initial funds to come up with a start-up. After our services offered to us, I went home, took a pen and paper and did a little calculation to see my way clear on printing press business. I was lucky, first, to secure container from my uncle free of charge, when I discussed the entrepreneur ideas with him. Within the same year, 2016, the price of container was going for (GH¢200.00, equivalent of US$46.73). I also searched for prices of printer (GH¢200.00, equivalent of US$46.73), desktop computer (GH¢800.00, equivalent of US$186.92), and laminating machine (GH¢400.00, equivalent of US$93.46), and binding machine (GH¢300, dollar equivalent of US$70.09) at the open market. I decided not to take a loan from any financial house for the micro-enterprise, and, therefore, talked to my uncle, aunts, nephews and friends. The initial funds of (GH¢3,500.00, equivalent of US$817.75) required for the business, over 60% were granted to me free, but the remaining 40% (sic) were to be paid back within four months without interest. It was a good start for me, and fortunately, I had committed clienteles. For instance, there was a private school very close to my shop, and not only the school, but the students also do most of their photo-copies at my shop. The school prints its exams questions at my printing press. By dint of hard work, and resilience, I was able to pay within a year the 40 percent of funds relations and friends lend to me. I have operated the printing shop for over five years now, and doing well.

Deducing from the story shared by the participant on *how* and *when* he commenced his entrepreneurial journey, one can say that the participant is a self-motivated person as Jones (2015), describes it. He persevered, endured and persisted in his entrepreneurial drive. Not having money to come up with a start-up, he went first to his uncle to explain to him what entrepreneurship is, and his interest in it. He managed to convince his uncle and sought for his container to commence his start-up. He then gained the boldness to move on, and received support from the family members and friends financialwise, to come up with his printing press. One can argue that the micro-enterprise was funded mainly by the extended family members, and that could impact positively, or negatively of management of the printing press. After all, in many African countries, a start-up or a business is a necessity to get an income as the participant case is, to enable him continue his education at tertiary level. Therefore, access to family financial support is an important source of capital to commence business, yet, such enterprises not only create opportunity for family members and relations to get jobs and receive incomes, but family responsibilities may also tap the enterprise for resources at later stage and makes it difficult to maintain the accumulated financial resources needed to develop the enterprise (Iguisi, 2003). A report from a study of selected European countries and Nigeria, by Iguisi (2003), indicated that the Nigerian respondents had many family members who depended on the business man for financial support. From the participant narrative, however, such a challenge did not show up in his printing press micro-enterprise, and that might have motivated the participant to work harder and make a niche in his identified line of business.

### Progress in micro-enterprise in the short-term

As to the performance of the micro-business in the past two years, the participant narrated the story and his experiences in this succinct manner:

I must say that within the first 2 years, I worked very hard to pay all the debt I owed my creditors, essentially family relations and friends who supported me to create the start-up. As I worked hard in the first two years to pay my creditors, eventually, the micro-enterprise is mine now, and therefore, has to push it hard to make a mark.

The participant story demonstrates that he knew that he was in business, and needs to work hard to drive his micro-enterprise on a right path. He did that and cleared the debts he owed his creditors. This support Knowles (2003), Cordeiro (2007), Murphy (2010), and Biney (2019) observation that plain hard work pays in entrepreneurship, especially in a developing country such as Ghana, where appropriate infrastructure and facilities to aid entrepreneurs to push their enterprises to the next level are woefully non-existent, or at best, inadequate. The participant, however, persevered, and worked to sustain his micro-enterprise. That is expected because, coming from a family of 16 people, comprising 11 children and five wives, and none of the 11 children has taken his education to tertiary level, the participant was determined to succeed. And he succeeds in both entrepreneurial drive and education; he could then extend support to the younger siblings to further their education. And with sound education, he was hopeful his micro-enterprise would thrive.

### Prospects of micro-enterprise in the medium term

When probed further on where participant intends to see his micro-enterprise in five years, he provided the narrative in this apt way:

In the first place, my first printing shop has travelled for over five years now. And, in fact, within the first five years, I saw my business grew because business was good. Hence, I opened two more printing shops and hired four workers. All of them were located closed to schools to secure more clienteles. The two printing shops were located in Accra, the capital of Ghana. I must also say that I have built a customer base for my first printing shop at Asare-Botwe in Accra, and I will do everything possible to maintain, and further grow not only my first micro-enterprise, but the additional two micro-enterprises. In actual fact, the locations of the printing shops account for their success, or otherwise. My next printing shop will be located close to a university with the hope of getting more clienteles patronising my services. This is because, as a student at Accra Learning Centre, whenever I go to the Centre, I see many colleagues patronising the services of the small printing shop located there. This gives me the hope that when I locate a printing shop in a university, I’m more likely to do a good business. I’m also of the view that some adult learners, in spite of increased online learning, prefer printing the learning materials and photo-copying past questions and try their hands on them. This is probably because some adult learners seem not to read extensively from online but from the printed materials. This is based on the fact that anytime I go to my Centre, I continue to see many adult learners printing, binding, and laminating learning materials for their private studies.

The participant seems observant, and can visualise, and has the entrepreneurial eye to spot an unrecognised need, or opportunity as he pointed out in his narrative. And this observation is supported by Cordeiro’s (2007) assertion that entrepreneurs have the entrepreneurial eye to identify a need or an opportunity in a community where ordinary people cannot identify and see needs to be exploited for job creation. The participant is maturing as an entrepreneur, and more likely to fit into Unger et al. (2011) categorisation of habitual entrepreneur. Ghana as a developing country needs many such young people with vision to help create sustainable jobs for the unemployed youth to do. This ties in well with Wright et al. (2022) assertion that entrepreneurship should be taught in the universities for all students to foster, nature and grow their entrepreneurial mindsets and create sustainable jobs for themselves and many others.

### State of micro-enterprise in the long-term

When the participant was asked the long term plan, that is, between seven to ten years’ time where does he wants to see his micro-enterprises, he indicated that he has plans for his micro-enterprises and narrated the plans in this best way:

There are plans in place to move my micro-enterprises a notch higher. In fact, I intend to stay within the printing shop business knowing very well how good I’m faring in this eco-system. If things work as planned, I intend to establish two more printing shops in addition to the three printing shops I have already established. However, I will place these two printing shops in strategic points or places, thus, close to schools and universities to ensure their survivability. I also intend to increase the workers from six to twelve, depending on the viability of not only the two new printing shops, but the already existing three printing shops. If the existing three printing shops do well as I see them doing now, I will continue to drive this micro-business. I’m also thinking about stocking the proposed two new printing shops with new and modern equipment on the market, to engender smart and efficient business. It is my hope that when I do my homework well, especially by actively marketing my business, I will succeed in my entrepreneurial drive.

This narrative recounted by the participant demonstrates that he has solid plans for growth of his micro-enterprises. He is learning to secure appropriate knowledge to booster his printing shop, thus, not only considering modern machines in printing shop, but also marketing services he renders to propel his micro-enterprises to a higher level. Ensign and Farlow (2016) have made us understand that an ecosystem helps entrepreneurs build social capital because of the abundance of individuals involved in entrepreneurship; and this is likely to lead to building human capital. This is good for entrepreneurship, and can lead to deepening entrepreneurial capital in Ghana. Again, one is tempted to believe that the participant is using both sides of his brain, thus, convergent and divergent thinking; doing logical thinking and reasoning, and also being imaginative backed by emotional processes. The participant is forward looking and has plans for his micro-enterprises. Creativity and innovation, to Chaniago (2022), Cordeiro (2007), and Yeung (2007) are keys to success in entrepreneurship. These are qualities that budding entrepreneurs should foster to promote and propel their micro-enterprises to a higher notch.

### Participation in NFE and informal programmes

On participation in NFE to revitalise and grow printing shops micro-enterprises, the participant presented his narratives, stories, and experiences in this apt way:

My participation in NFE and informal learning has aid me to drive the progress of my micro-enterprises. Hitherto, I wasn’t in the position to design products, use the web successfully, use excel and become proficient in other skills to propel my investment in printing shop enterprise. I have been participating in workshops and seminars, but yet to participate in management boot-camps. What I have been doing more to move my business forward was, and still is, learning informally online. I go online a lot and learn more about the printing business, and even learn about how to source for new machines for my micro-enterprises. Indeed, it is the online exploration which has aid me greatly in my printing shop business. In the case of participation in management boot-camps, I have seen a number of advertisements online, but yet to take advantage of them. Meanwhile, I’m aware that acquisition of solid managerial talent, skills and knowledge can propel me push my micro-enterprises a notch higher. I’m strategising to participate in the next management boot-camps training programme that comes to my notice. It is my firm conviction that whatever knowledge and skills that I will acquire can help me further grow my micro-enterprises.

From this narrative, one can deduce that the participant was desirous to learn formally, informal and non-formally, thus adopting a lifelong learning approach to grow his micro-enterprises. Apart from lecture hall learning, people in today’s digital era learn over the course of their lives, and I find it a desirable thing to do. It was therefore not surprising that the participant engaged in lifelong learning, be it exploration online, participating in seminars, symposia, workshops to grow his micro-enterprises. Considering it from an informal learning angle, Tusting and Barton (2006) opine that informal learning is often necessary to do the job; and the participant did learn to grow his micro-enterprises. Skills and talent in management is critical to enterprise growth (Abor & Quartey, 2010), the participant observed that and agreed to take advantage of it in the next available time. This observation is important to the extent that the lack of managerial talent among entrepreneurs and managers led to the collapsed of many enterprises established in the 1960s and 1970s in Ghana (Biney, 2019). This resultant effect is the mass extensive unemployment confronting young adults in Ghana today.

### Preferred NFE/informal programmes to grow micro-enterprise

Probed further which of the NFE programmes could aid the participant to grow his micro-enterprises sustainably, he took the narrative to another level:

My participation in management boot-camps could *do the trick* because I’m already managing three printing shops, therefore, with updated knowledge and skills in micro-enterprises, I can become successful in my businesses. As a human resource management student, the knowledge from such training programmes can put me in a better stead to manage both my micro-enterprises and personnel for betterment than I’m currently doing. Hence, I need more managerial talent, knowledge and skills to be successful in my entrepreneurial drive.

The participant has come to agree that skills and knowledge in management is key to successful growth of his micro-enterprises. He is a student of human resource management and being taught now via an online learning approach, but some capacity building programme in the form of a management boot-camp could help the participant comes to terms with practical approaches to managing his growing printing shops. Abor and Quartey (2010) identified the lack of managerial skills as a key challenge to successfully drive small and medium enterprises on a sustainable path in developing countries. This observation requires attention if young adults are to succeed in entrepreneurship.

### Challenges and coping strategies

Asked whether the participant’s micro-enterprises have experienced challenges and which coping strategies were adopted, he provided the narratives, stories, and experiences in this way:

Yes, I have witnessed challenges in my micro-enterprises, but the biggest has been the Covid-19 pandemic. The Covid-19 crisis has really been my *‘achilles heel’* in my micro-enterprises, especially in the year 2020. It became extremely difficult for my ventures because my reliable clienteles, including schools and students, and pupils in Junior High School (JHS) were all at home as schools closed down as a result of the devastating effect of the Covid-19 pandemic. It hugely affected my stream of income from the ventures. The next challenge faced has to do with machines used in my printing shops. When there is a break down in some of the machines, sometimes the replacement parts could even buy a whole new machine on the market. The prices usually quoted for parts to be replaced are costly. However, the improvement being witnessed in the Covid-19 situation in Ghana has led to a gradual improvement in business. My plans still hold that by the end of the year 2021, I hope to establish two more printing shops in Accra. On coping strategies adopted, I seriously embarked upon savings culture when business was good, therefore, the Covid-19 pandemic difficulties though devastating, I survived it. Again, the pieces of advice provided by friends and my bankers helped me, coupled with hard work, have placed me in where I’m today. Indeed, hard work is the name of the game. Currently, I’m financing my education at the University of Ghana.

Deducing from the narrative presented, it can be said that the printing shop business is rewarding, but also involves cost. It is not surprising that the participant cultivated a saving culture as a measure of cushioning his micro-enterprises. Recounting on his ventures, he indicated that the Covid-19 crisis has been his *‘achilles heel’,* thus the biggest challenge faced by his micro-enterprises. This observation resonates with Chaniago’s (2022) assertion that it is challenging to run a business during the Covid-19 pandemic because the owner and the small business must be responsible. Though the participant witnessed difficulties during the Covid-19 crisis, he was responsible, and therefore cushioned his micro-enterprises preventing them from possible collapse. He exhibited plain hard work, resilience and tenacity of purpose to drive his micro-enterprises. This should be a lesson for budding entrepreneurs to cultivate those qualities, including the culture of savings. That said, however, Covid-19 was not only a problem for enterprises, but individuals, communities, families, institutions and organisations (Smith et al., 2021). The participant further indicated that parts for broken down machines are expensive and costly. This probably led the participant to build a savings culture such that he could, at any point in time, have the necessary wherewithal to procure new parts to fix broken down machine(s).

## Discussions

The study explored adult education and entrepreneurship and sought ways of involving young adults to become entrepreneurial and foster growth and enterprising mindset. The researcher saw a gap, noting that adult education which relates to entrepreneurship has received little research, although both fields have everything to do with learning. The researcher felt that when young adults participate in adult education programmes as a precursor to entrepreneurship, they could exploit opportunities in their communities, foster entrepreneurial mindsets and become self-employed.. Entrepreneurial learning has never been more important than today (Lindner, 2020); based essentially on the growing youth population, and rising unemployment in many countries, including Ghana. Additionally, according to Lindner (2020), changes in the labour market and technological developments, are some of the reasons why we must provide the future generations with entrepreneurial skills and mindsets they need to succeed in the twenty-first century.

On fostering entrepreneurial ideas and mindset, it emerged that the participant possesses a growth and enterprising mindset and this finding confirms the observations of Knowles (2003), Jones (2015) and Dweck (2008, 2016) that entrepreneurs should be self-motivated and committed to their course of action. This finding contrasts with Schermerhorn (2005) observation about insufficient commitment of young adults to their enterprises, contributing to failures of youth enterprises. In the case of the participant, he showed sufficient commitment to his printing shops and even survived during the peak period of the COVID-19 crisis. The participant took a calculated and informed risk of investing in printing shops or micro-enterprises. He was ready to learn so he sought for information on the printing shop business from the manager of the printing shop he visited with the friend. The participant also explored online for rich information on managing a printing shop. Resorting to the family first for financial support for the micro-enterprise is the best in the sense that when the business fails, as over 70% of newly created micro-enterprises do (Cordeiro, 2007); the problem could be addressed within the family, rather than resorting to legal redress usually associated with credit received from the commercial banks. The participant developed for himself the desire to work hard. He also exhibited grit, ingenuity, and entrepreneurial responsibility (Takyi-Appiah, 2022). He learned to make a mark in his micro-enterprises and the entire society, by creating job opportunities for himself and others. This hard work and ‘self-made’ approach to success should be cultivated by young adults, rather than resorting to short-cut approach to success via ‘galamsey’. This is because, this illegal and dangerous activity has created huge challenges to the economy of Ghana in terms destruction of water bodies and degradation of the environment.

In terms of progress in micro-enterprise in the short-term, the participant demonstrated that he was determined to work to take care of himself and continue his education. Within the first 2 years, he worked hard to pay all his debts he owed creditors who cushioned him to come up with the printing shop. Knowles (2003), Cordeiro (2007), Yeung (2007), and Murphy (2010) opine that hard work is a critical ingredient to success in entrepreneurship. The result also ties in well with Nafukho and Muyia’s (2010) observation that students who learn entrepreneurship have learned to be creative and innovative, and should seek to be employment creators and not job seekers. Although the participant is not reading entrepreneurship as a course, informally, he learned and practiced entrepreneurship and created job opportunities for himself and others. The participant exhibited unique characteristics to grow his printing shops. The plain hard work exhibited accounted for the successful management of his micro-enterprises.

On prospects of micro-enterprise in the medium term, it was refreshing to learn from the participant narratives that he worked extra hard to create two more printing shops in strategic places in Accra, and hired four people. It became clear that the printing shops were also sited close to schools. That is understandable to the extent that, that is where the participant has his clienteles- schools and students doing photo-copies of examination questions, past questions to guide learning, binding and laminating of printed works. The intention of the participant to grow his enterprises and create job opportunities for many young adults is a laudable idea. Again, unemployment has been the bane of young adults, especially in the developing countries, including Ghana, so when young adults are encouraged and motivated to go into entrepreneurship as Ofori-Atta (2021) argued, then, it is a well thought out idea which should inform policy decision making on employment issues, particularly, in Ghana. The public sector cannot employ many people, therefore, adult education and entrepreneurship should vigorously be promoted amongst young adults to explore and create ventures for themselves.

On participation in NFE and informal programmes, the study revealed that aside from formal education, the participant also engaged in informal exploration on the web to learn more about printing shop micro-enterprise. The participant participated in seminars and workshops on entrepreneurship, yet failed to take advantage of management boot-camps. In fact, Abor and Quartey (2010) assert that managerial talent, skills, and knowledge in enterprise have been the bane of many entrepreneurs managing micro, small and medium enterprises (MSMEs) in the developing countries. The result also resonates with Dweck’s (2016) observation that in many instances, entrepreneurial characteristics and skills can be learned. As revealed by Zappe (2019), people with growth mindsets have certain characteristics that with *training* and *practice* we can improve and change those characteristics. No wonder, the participant which can be described as a person with growth and enterprising mindsets, indicated that he would take advantage of management boot-camps at the next opportunity. More so, today’s digital era in the global economy, enables people who engage in lifelong learning to be successful in the workplace (King, 2017; The Economist, 2017). Therefore, when young adults are cushioned and motivated to engage in lifelong learning and dare to venture into entrepreneurship, much of the issue of graduate unemployment could be addressed in our part of the world.

On preferred NFE/informal programmes to grow micro-enterprises, the participant place more emphasis on online exploration, thus, learning informally. The Internet has made the world become a global village, and people can learn more about their professions from the web. Indeed, lifelong learning is, today, the name of the game. In addition, participating in hands-on practical training in management boot-camps is important, especially in the case of micro-enterprises where over 70% (Cordeiro, 2007) are prone to failure and collapse. The participant, and for that matter, young adults who have the desire and interest in entrepreneurship must make adult education and lifelong learning their pillars in their micro-enterprises.

On challenges and coping strategies adapted by the participant, the study indicates that the participant faced challenges in growing his micro-enterprises, yet he prevailed. The biggest of the problems has to do with the COVID-19. This pandemic made it nearly impossible to attract his clients to his shop to patronise his services. What saved him and his business, according to the participant, is the savings culture that he cultivated as a strategy to cushion the growth of his printing shop business. Although Archer and Newman (2003) observe that two reasons for failure of micro-enterprises in developing countries are first, not enough attention paid to the macro-environment, and second, micro-enterprises operators are not supported within a wider *learning* process with skills for business accounting and record keeping. Legodi and Kanjere (2015) also identified lack of skills as a challenge facing majority of enterprise promoters. Amadasun and Mutezo (2022) indicated that lack of access to financial or credit information and support from banks constitute some of the challenges that confront micro-enterprises. In this study, however, the participant *learned* to build a strong savings culture, so he weathered the storm, posed to his business by the COVID-19 pandemic. Again, the prices quoted for parts of machines used for printing shop were costly, yet the participant managed to operate his printing shop, and made some revenue and created additional two more printing shops. The participant still has a plan to create two more printing shops in the long term to create additional jobs for young adults. The human capital theory is still relevant, and when properly applied, especially in the developing countries, young adults could become proactive to unleash the potential of entrepreneurship to make their lives and many others better. A case study in China referred to as ‘Mass Entrepreneurship and Innovation’ initiative as part of national development strategy suggested fostering entrepreneurship amongst young adults. This initiative required universities to increase resources for entrepreneurial activities and for *all students* to complete an entrepreneurial course (Wright et al., 2022). The fact too is that such an entrepreneurial education not only aids those who start their own businesses but also each student can take notions of innovation and enterprising mindset built into them to their workplace employment in order to help the organisations of others to develop and flourish. In fact, Talmage and Gassert (2020) illuminated on the importance of social entrepreneurship which students should begin to learn and acquire the requisite skills and knowledge to be relevant at their workplaces. In the Chinese situation, the universities demonstrated an economic contribution to the public good by instilling students with entrepreneurialism and providing guidance for starting businesses (Wright et al., 2022). The Ghanaian ‘YouStart’ model meant for building an entrepreneurial state does not provide all students with entrepreneurial education, but endeavours to cushion graduates from tertiary institutions with some funding to commence start-ups. This is where the problem starts. In my view, every young adult must first receive some formal and NFE education on entrepreneurship at the universities, and then build on it in the world of work with more NFE and informal education and training. I argue here that if young adults’ interests in entrepreneurship are nurtured at the university level as the Chinese have been doing, young adults in Ghana would have been empowered to initiate, grow and manage their micro-enterprises successfully.

## Implications

Entrepreneurship and adult education are receiving attention in developing countries in particular, because they possess the potential to making the many unemployed young adults become self-employed and, thus, job creators rather than job seekers. No wonder, entrepreneurship has long been made an explicit policy priority (Lind, 2012). Lindner (2020), Rasmussen (2014) and Debyser (2013) assert that the European Union (EU) indicated that *adult education* has a strong link to work and the labour market, and the “New Skills for New Jobs initiative” adapted by EU improves employment by supporting *entrepreneurship* and *self-employment*. Entrepreneurship, though well practiced in the Western world, especially the U.S. where Jones (2015) argues that many people foster an enterprising and growth mindset, and have high need for self-fulfillment, and possess passion to create something for others to benefit. Entrepreneurs pursue innovative ideas, and ensure that they become reality even when things get in the way. Entrepreneurs are self-motivated individuals and work hard to turn difficult situations around, and this is what Kourdi (2015) describes as pragmatism in thinking and action. The point here is that as young adults engage in adult education, and for that matter lifelong learning, and what Lindner (2020), and Lackeus (2015) refers to as *entrepreneurial learning,* they are able to turn their lives around through fostering entrepreneurial mindset to create start-ups for themselves.

The Government of Ghana (GoG), for instance, has put in place a National Micro, Small and Medium Enterprises and Entrepreneurship (MSMEs) Policy. It is hoped that when it is operationalised, young adults would benefit, grow and make their enterprises sustainable to create job opportunities for unemployed young adults in Ghana. The reality though is that many young adults would like to engage in entrepreneurship, but need appropriate entrepreneurship training, advisory, technical and financial resources as is the case in China, to be able to do so. The existing entrepreneurship agencies, including National Entrepreneurship and Innovation Plan (NEIP), Ghana Enterprises Agency (GEA) Youth Enterprise Agency (YEA), and Micro-Finance and Small Loan Centre (MASLOC) have not been able to provide the much needed financial, technical and advisory support required by adventurous young adults who want to go into entrepreneurship. Currently, the GoG is implementing ‘YouStart’ programme, another entrepreneurial intervention with GH¢10,000,000.00, equivalent of US$1,666,666.67 investment amongst young adults to build an entrepreneurial state. This laudable intervention required support in the form of access to capital, training, technical skills, advisory and mentoring services provided to young adults at the right time to build entrepreneurial mindsets.

It is therefore imperative that facilitators and lecturers on the DE mode of learning encourage young students to explore more in their communities to identify and spot unrecognised needs and exploit them to create jobs for themselves. But this can only happen when entrepreneurship education, training and practice is duly incorporated into the curriculum of higher education institutions in the developing countries. When internships and attachments are encouraged by departments in the universities with corporate organisations, young adults would become empowered to venture into entrepreneurship. Sernau (2009) observes that learning needs to lead to internships, apprenticeships, and other opportunities for its useful application. Hence, there should be regular seminars and workshops instituted and labeled as *“Time with Captains of Business”* to offer talks on their enterprising journey, and how they navigated the entrepreneurship terrain to become astute entrepreneurs. These programmes possess the potential to motivate and stimulate adventurous students to think creatively and foster entrepreneurial mindset in their communities, where entrepreneurial opportunities abound.

## Conclusions

This exploratory study probed into adult education and entrepreneurship and how young adults could get involved. A level 100 adult learner aged 26 years reading Human Resource Management, Psychology and Information Studies served as the unit of analysis. Seven probing questions were posed to the participant, and the overall results demonstrate that the participant fostered growth and enterprising mindsets. He worked hard and chalked successes in entrepreneurial drive. He worked to pay his university education fees and created jobs for six people. The participant planned to create two more printing shops to the three already existing in the long term, and create jobs for twelve people. An example of an enterprising young adult whose capacities has to be built through adult education and entrepreneurship programmes. Though the participant faced challenges in growing his micro-enterprises, the biggest was Covid-19, but the savings culture cultivated made him prevail against all odds. This is a qualitative study and the results cannot be generalised. I suggest that a quantitative study is conducted on motivational strategies to promote entrepreneurship amongst young adults.

The study recommends that key stakeholders in entrepreneurship, including governments, should by way of policy and HEIs by way of curriculum reform, facilitation and partnership with the private sector, build capacities of young adults and support them with financial and advisory services to go into entrepreneurship. By so doing, young adults would learn to take moderate and informed risks to invest and create sustainable micro-enterprises. Young adults then would not only become self-employed in their enterprises, but create job opportunities for the many unemployed young adults in Ghana.

Considering the findings and recommendations made, this study has implications for adult education and entrepreneurship theories, policies and practices. The study has managerial implications too. This is an inter-disciplinary study, and theoretically, it reinforces connections between adult education and entrepreneurship, and contributes to existing literature on human capital theory, capability theory, quadruple and quintuple innovation helix in adult education and entrepreneurship. Both fields involve learning, which is a critical component to succeed in today’s digitally dominated global knowledge economy. People, today, have the capacity to learn and become creative to transform their lives, communities and societies at large if they learn to identify opportunities and exploit them. The researcher suggest that further studies are conducted to impact on theories of adult education, emphasising on fostering the adult lifelong learning drive, and entrepreneurship, focusing on creativity and innovation of human beings. Managerially, this study illuminates that any policy intervention made for young adults to become self-employed should be properly thought through, managed, monitored, assessed and evaluated to achieve the intended results. Lack of effective management of industries created in the 1960s and 1970s in Ghana collapsed, signifying that learning to secure managerial talent is crucial in the survival of enterprises. This demonstrates that the capacity and organisational acumen of young adults are regularly built through workshops and management boot-camps to become savvy and versatile in entrepreneurship. Similarly, the environment in which young adults learn and apply their skills should be conducive and inviting, to attract their attention and action.

In practice, more hands-on training, mentorship, internship and apprenticeship should be encouraged and provided to young adults to improve knowledge, skills and positive attitude toward entrepreneurship. When this is done, many young adults would become self-confident, resilient, and build the entrepreneurial spirit to create ventures for themselves. They will learn to build financial literacy skills, use purposeful searching to engage in careful planning, and make sound judgement whenever they are carrying out entrepreneurial activities.

## Data Availability

An in-depth guide was designed under the heading ‘Adult Education and Entrepreneurship: Getting Young Adults Involved’, to collect data from the participant for the study. The said instrument is attached on a separate document.
